# Supplementary material for: Prevalence, patterns, and determinants of multimorbidity in South Africa: Insights from a nationally representative survey
Source: PLOS Glob Public Health. 2025 May 9;5(5):e0004603. doi: 10.1371/journal.pgph.0004603 (PMC12063843; doi:10.1371/journal.pgph.0004603)
Supplement: S1 Table — (DOCX) [file pgph.0004603.s001.docx]

**S1 Table. Definitions of outcome measures**

| **Outcome measure** | **Definition** |
| --- | --- |
| *Objective/ biomarker-measured measures* |  |
| Hypertension | Digital blood pressure monitors were used to take three blood pressure measurements, at intervals of three minutes or more [1]. As per convention, the study excluded the first blood pressure measurement and utilised an average of the remaining two measurements [2]. Hypertension was classified as individuals who have either a systolic blood pressure of ≥140 mm Hg or a diastolic blood pressure of ≥90 mm Hg [3]. People with non-hypertensive biomarkers who self-reported that they were on medication to treat hypertension were recorded as hypertensive. Implausible biological marker values were recoded as missing (SBP <69 or >271 mm Hg, DBP <29 or >151 mm Hg). |
| Diabetes | Dried blood spots analysed at the Global Clinical and Viral Laboratory in Durban were used to record diabetes and individuals with HbA1c ≥ 6.5 mmol recorded by a blood chemistry analyser were classified as diabetic, as were those with healthy HbA1c values who self-reported taking diabetes medication. |
| Anaemia | For anaemia, non-pregnant women, pregnant women, and men with haemoglobin levels below 7 g/dl, 9g/dl and 9 g/dl were classified as anaemic, respectively [1]. Anaemia testing was carried out on site and results were adjusted for smoking status and altitude. |
| HIV | Dried blood spots analysed at the Global Clinical and Viral Laboratory in Durban were used to record HIV. An enzyme-linked immunosorbent assay (ELISA) was used to test for HIV, a second ELISA and an alternative confirmatory rapid test (Bio-Rad) were used to confirm the initial ELISA test. Individuals were either classified as HIV positive, HIV negative or inconclusive, those who were inconclusive were recoded as missing. |
| *Self-reported measures* |  |
| High blood cholesterol  Heart attack  Asthma  Chronic bronchitis  Tuberculosis  Stroke  Cancer  Chronic pain | Participants were asked ‘Has a doctor, nurse or health worker told you that you have or have had any of the following conditions: heart disease?’, with participants responding either ‘Yes’, ‘No’ or ‘Don’t know’ [1]. A response of ‘Don’t know’ was recoded as ‘No’. Individuals who self-reported a disease did not always concurrently report taking medication for the disease. To ensure diseases were chronic, tuberculosis had to have occurred within the previous 12 months and chronic pain had to last longer than three months; these were confirmed with responses to further questions. |

References

1. National Department of Health, Statistics South Africa, South African Medical Research Council, ICF. South Africa Demographic and Health Survey 2016. Pretoria, South Africa; 2019.

2. Roomaney RA, van Wyk B, Cois A, Wyk VP Van. One in five South Africans are multimorbid: An analysis of the 2016 demographic and health survey. PLoS One. 2022;17: e0269081. doi:10.1371/JOURNAL.PONE.0269081

3. World Health Organisation. Hypertension - Fact Sheet. 16 Mar 2023 [cited 23 Jan 2024]. Available: https://www.who.int/news-room/fact-sheets/detail/hypertension
