# Supplementary material for: Prevalence, patterns, and determinants of multimorbidity in South Africa: Insights from a nationally representative survey
Source: PLOS Glob Public Health. 2025 May 9;5(5):e0004603. doi: 10.1371/journal.pgph.0004603 (PMC12063843; doi:10.1371/journal.pgph.0004603)
Supplement: S2 Table — (DOCX) [file pgph.0004603.s002.docx]

**S2 Table. Information on explanatory variables and their derivation**

| **Variable** | **Information** |
| --- | --- |
| Wealth index | Wealth index was characterised by DHS using principal component analysis of ownership of various household goods and other characteristics (i.e., type of flooring; source of water; availability of electricity; possession of durable consumer goods), split into quintiles (National Department of Health et al., 2019). For more information on this and other variables see: <https://www.dhsprogram.com/publications/publication-fr337-dhs-final-reports.cfm?cssearch=16620_1> |
| Education level | Educational level was categorised into the highest educational level achieved, split into no education, primary-level education, secondary-level education, and higher-level education. Derived from ‘Highest education level’ variable. |
| Occupational status | Occupational status was split into eight categories: unemployed, professional/technical/management, clerical, agricultural, domestic services, sales and services, skilled manual, and unskilled manual. From the initial DHS categorisation ‘agriculture-self-employed’ and ‘agriculture – unskilled’ were combined, as were ‘household and domestic’ and ‘services’ to ensure sufficient data within categories. Derived from ‘Respondent currently working’ and ‘Respondent occupation (grouped)’ variables. |
| Health insurance | Whether individuals were covered by health insurance, yes/no (Y/N). Those missing information were coded as negative. Derived from ‘Covered by health insurance’ variable. |
| Marital status | Whether individuals were never in a union (married or living with partner), are currently in a union, or formerly in a union (widowed/ divorced/ separated). Derived from ‘Current marital status’ variable. |
| Age category | Age was categorised into ten-year bands from 15 to 64, and all those aged above 65 were banded together. Derived from ‘Current age’ variable. |
| Sex | Whether individuals were male or female. Derived from ‘Sex’ variable. |
| Ethnicity | Ethnicity categorised into black/African, white, mixed ancestry, and Asian/other. Derived from ‘Ethnicity’ variable. |
| Body mass index (BMI) | A digital scale and stadiometer were used to measure height and weight. According to WHO guidelines, BMI was categorised as follows: underweight as <18.5kg/m2, healthy weight between 18.5 and 24.9 kg/m2, overweight as between 25.0 and 29.9 kg/m2, and obesity as >30 kg/m2 (World Health Organisation, 2020). Women pregnant or who had given birth within two months were coded as missing, as BMI was not possible to ascertain. Derived from ‘Body Mass Index’, ‘Last birth to interview (months)’ and ‘Currently pregnant’ variables. |
| Dietary health | Six questions on dietary health were divided into four categories, combined together and split into low, medium and high categories. Derived from ‘frequency eat fried foods’, ‘frequency eat fast foods’, ‘frequency eat packed chips’, frequency east processed meat’, ‘types of fruit eaten yesterday’, and ‘types of vegetables eaten yesterday’ variables. |
| Sugary drink intake | Five questions on sugary drink intake were combined and split into three categories (low, medium, high); ‘Number of sugary drinks yesterday’, ‘Low amount of sugary drinks yesterday’, and ‘High amount of sugary drinks yesterday’. Derived from ‘sugar-sweetened drinks yesterday’, ‘number of sugar-sweetened drinks of 200ml glass’, ‘number of sugar sweetened drinks of 330ml can or bottle’, ‘number of sugar-sweetened drinks of 500ml bottle’, ‘number of sugar-sweetened drinks of 1L bottle’ and ‘number of sugar-sweetened drinks of 2L bottle’ variables. |
| Smoking status | 13 variables combined to determine whether individuals were current smokers (smoke manufactured cigarettes, hand-rolled cigarettes, pipe of tobacco, cigars and more tobacco-based products daily, weekly or less than weekly), ex-smokers (if smoked in the past but not currently) and never smokers. Derived from ‘frequency currently smokes tobacco’, ‘frequency in the past smoked tobacco’, ‘on average respondent smokes daily: manufactured cigarettes’, ‘on average respondent smokes daily: hand roll cigarettes’, ‘on average respondent smokes daily: pipes full of tobacco’, ‘on average respondent smokes daily: cigars, cheroots, cigarillos’, ‘on average respondent smokes daily: water pipe sessions’, ‘on average respondent smokes daily: others’, ‘on average respondent smokes weekly: manufactured cigarettes’, ‘on average respondent smokes weekly: hand roll cigarettes’, ‘on average respondent smokes weekly: pipes full of tobacco’, ‘on average respondent smokes weekly: cigars, cheroots, cigarillos’ and ‘on average respondent smokes weekly: water pipe sessions’ variables. |
| Alcohol drinking | Two questions were combined to determine whether individuals had drunk alcohol in the past 12 months (Y/N). Derived from ‘ever consumed alcohol’ and ‘consumed alcohol in last 12 months’ variables. |
| Exposure to smoke at work | Whether individuals were exposed to smoke at work (Y/N). Derived from ‘ever worked in a place exposed to smoke’ variable. |
| Access to old media | Responses to three questions on frequency of watching television, listening to radio, and reading newspaper or magazine, were combined and split into low, medium and high categories. Derived from ‘frequency of reading newspaper or magazine’, ‘frequency of listening to radio’ and ‘frequency of watching television’ variables. |
| Access to new media | Responses to two questions on whether owns a mobile phone and frequency of internet use in the preceding month were combined and split into low, medium and high categories. Derived from ‘frequency of using internet last month’ and ‘owns a mobile phone’ variables. |
| Neighbourhood-level poverty | Neighbourhoods were defined as respondents from clusters of households which serve as the PSU within the DHS. Defined as the proportion of individuals living in poverty for each neighbourhood. This was split into three categories (low, medium and high) with low as the reference group and calculated with the larger adult-health sample (N= 9,512) to include more contextual information. Derived from ‘Wealth index’ variable. |
| Neighbourhood-level rurality | Neighbourhoods were defined as respondents from clusters of households which serve as the PSU within the DHS. Defined as whether a neighbourhood resided in an urban or rural area and calculated with the larger adult-health sample (N= 9,512) with urban as the reference group to include more contextual information. Derived from ‘place of residence’ variable. |
| Neighbourhood-level illiteracy | Neighbourhoods were defined as respondents from clusters of households which serve as the PSU within the DHS. Defined as the proportion of individuals illiterate in each neighbourhood. This was split into three categories (low, medium and high) with low as the reference group and calculated with the larger adult-health sample (N= 9,512) to include more contextual information. Derived from ‘literacy’ variable. |
| Neighbourhood-level unemployment | Neighbourhoods were defined as respondents from clusters of households which serve as the PSU within the DHS. Defined as the proportion of individuals unemployed in each neighbourhood. This was split into three categories (low, medium and high) with low as the reference group and calculated with the larger adult-health sample (N= 9,512) to include more contextual information. Derived from ‘Respondent currently working’ and ‘Respondent occupation (grouped)’ variables. |
| Provincial-level poverty | Defined as the proportion of individuals living in poverty for each province. This was split into three categories (low, medium and high) with low as the reference group and calculated with the larger adult-health sample (N= 9,512) to include more contextual information. Derived from ‘Wealth index’ variable. |
| Provincial-level rurality (low as ref) | Defined as the proportion of individuals living in rural areas for each province. This was split into three categories (low, medium and high) with low as the reference group and calculated with the larger adult-health sample (N= 9,512) to include more contextual information. Derived from ‘place of residence’ variable. |
| Provincial-level unemployment (low as ref) | Defined as the proportion of individuals unemployed for each province. This was split into three categories (low, medium and high) with low as the reference group and calculated with the larger adult-health sample (N= 9,512) to include more contextual information. Derived from ‘Respondent currently working’ and ‘Respondent occupation (grouped)’ variables. |
