# Supplementary material for: Prevalence, patterns, and determinants of multimorbidity in South Africa: Insights from a nationally representative survey
Source: PLOS Glob Public Health. 2025 May 9;5(5):e0004603. doi: 10.1371/journal.pgph.0004603 (PMC12063843; doi:10.1371/journal.pgph.0004603)
Supplement: Attachment — Submitted filename: 06_ResponsesToComments.docx [file pgph.0004603.s004.docx]

|  | Comment | Suggest response |
| --- | --- | --- |
| 1 | 1. Your current Financial Disclosure states, “MH (Pre-doctoral Fellowship; NIHR302654) is funded by the National Institute for Health Research (NIHR). The views expressed in this publication are those of the author(s) and not necessarily those of the NIHR, NHS or the UK Department of Health and Social Care.”. However, your funding information on the submission form is missing. Please indicate by return email the full and correct funding information for your study and confirm the order in which funding contributions should appear. Please be sure to indicate whether the funders played any role in the study design, data collection and analysis, decision to publish, or preparation of the manuscript. | Thank you for pointing this out. We have now provided the full and correct funding information via email. The funding statement remains unchanged, as it accurately reflects the funding source and its role. The funders did not play any role in the study design, data collection and analysis, decision to publish, or preparation of the manuscript. |
| 2 | 2. Thank you for uploading your study's underlying data set. Unfortunately, the repository you have noted in your Data Availability statement does not qualify as an acceptable data repository according to PLOS's standards.    At this time, please upload the minimal data set necessary to replicate your study's findings to a stable, public repository (such as figshare or Dryad) and provide us with the relevant URLs, DOIs, or accession numbers that may be used to access these data. For a list of recommended repositories and additional information on PLOS standards for data deposition, please see [https://journals.plos.org/plosone/s/recommended-repositories](https://eur01.safelinks.protection.outlook.com/?url=https%3A%2F%2Fjournals.plos.org%2Fplosone%2Fs%2Frecommended-repositories&data=05%7C02%7Colalekan.uthman%40warwick.ac.uk%7C79d7a361190542d9cd2f08dcf4ead689%7C09bacfbd47ef446592653546f2eaf6bc%7C0%7C0%7C638654540085708320%7CUnknown%7CTWFpbGZsb3d8eyJWIjoiMC4wLjAwMDAiLCJQIjoiV2luMzIiLCJBTiI6Ik1haWwiLCJXVCI6Mn0%3D%7C0%7C%7C%7C&sdata=evNOeahMbGYowvRQWGyUU9wVgu3GWaiwI0QSs9LCtiM%3D&reserved=0). | We have now uploaded the minimal dataset to Figshare a stable and public repository that meets PLOS's standards. The dataset can be accessed via the following link: ( <https://doi.org/10.6084/m9.figshare.28424369>). This link has been added to the Data Availability statement in the manuscript. |
| 3 | 3. Please provide separate figure files in .tif or .eps format.  For more information about figure files please see our guidelines:  [https://journals.plos.org/globalpublichealth/s/figures](https://eur01.safelinks.protection.outlook.com/?url=https%3A%2F%2Fjournals.plos.org%2Fglobalpublichealth%2Fs%2Ffigures&data=05%7C02%7Colalekan.uthman%40warwick.ac.uk%7C79d7a361190542d9cd2f08dcf4ead689%7C09bacfbd47ef446592653546f2eaf6bc%7C0%7C0%7C638654540085730963%7CUnknown%7CTWFpbGZsb3d8eyJWIjoiMC4wLjAwMDAiLCJQIjoiV2luMzIiLCJBTiI6Ik1haWwiLCJXVCI6Mn0%3D%7C0%7C%7C%7C&sdata=9rW6X8PFcg8A1UI8rn%2FWSOmiIQQ%2BAGs%2FewS%2FL6R5d6k%3D&reserved=0)  [https://journals.plos.org/globalpublichealth/s/figures#loc-file-requirements](https://eur01.safelinks.protection.outlook.com/?url=https%3A%2F%2Fjournals.plos.org%2Fglobalpublichealth%2Fs%2Ffigures%23loc-file-requirements&data=05%7C02%7Colalekan.uthman%40warwick.ac.uk%7C79d7a361190542d9cd2f08dcf4ead689%7C09bacfbd47ef446592653546f2eaf6bc%7C0%7C0%7C638654540085746618%7CUnknown%7CTWFpbGZsb3d8eyJWIjoiMC4wLjAwMDAiLCJQIjoiV2luMzIiLCJBTiI6Ik1haWwiLCJXVCI6Mn0%3D%7C0%7C%7C%7C&sdata=6R10ZcTMNGKKh%2BPJEYEg6XH3Jo0UX%2FdPiwBjMWBSqIc%3D&reserved=0) | We have now provided all figures in publication-quality .tif format and uploaded them separately as required. |
| 4 | 4. We notice that your figures are included in the manuscript file. Please remove them as there are already an uploaded file. | All figures have been removed from the manuscript file and are now uploaded separately. |
| 5 | 5. We have noticed that you have uploaded Supporting Information files, but you have not included a list of legends. Please add a full list of legends for your Supporting Information files after the references list. | We have now added a full list of legends for all Supporting Information files after the references list. |
|  | Reviewers' comments: |  |
| 6 | Reviewer #1: First, I would like to thank the editorial board for the opportunity to review this article. I consider it to be a highly interesting work, wherein the authors estimate the prevalence and distribution of multimorbidity in South Africa, as well as determine the risk factors associated with the presence of multimorbidity. I find the use of multilevel models particularly intriguing, although I have some doubts, comments, and suggestions. I am uncertain whether it is necessary to mention the type of adjusted model (multilevel analysis) in the title. Although multilevel analysis is not widely used, it is not a novelty either. This is, however, a purely personal opinion. | We agree with the reviewer’s suggestion and have removed the mention of “multilevel analysis” from the title. |
| 7 | I couldn't analyze the bibliographic references because in the copy I received, they appear numbered in order of appearance within the article's text, while in the bibliography section they are listed alphabetically. | We apologize for this inconsistency. We have now reformatted the references to follow the PLOS ONE format, with references numbered sequentially in the order of appearance in the text. This ensures consistency and clarity. |
| 8 | The introduction appears to be very well-written, clearly describing the objectives of the work and its potential implications. | We thank the reviewer for their positive feedback on the introduction. |
| 9 | MATERIALS AND METHODS  Study population  I suggest referencing Figure 1 (Study population). | We have removed Figure 1 (Study population) as it was not essential to the manuscript. The study population is now described in detail in the text. |
| 10 | Outcome variable  On page 7, lines 137-138, the 12 diseases from which multimorbidity is identified are mentioned (repetitively), having just been presented in the previous paragraph (page 6, lines 128-131). | We have removed the repetitive mention of the 12 diseases in the Outcome Variable section. The list is now presented only once for clarity. |
| 11 | On page 7, lines 139-141, the authors provide an example of a question and answer from the form. I find it more interesting to show the form in the supplementary material and reference it there, at least regarding the questions related to diseases. | We agree with the reviewer’s suggestion and have moved the detailed definitions of outcome measures, including the example question and answer, to the Supplementary Material. The relevant section in the manuscript now references the supplementary file for further details. |
| 12 | On page 7, line 142, the authors state that if the interviewee responds that they don't know if they have a disease, this is interpreted as not having it. I find this criterion inappropriate. Rather, they should treat it as missing data, exactly as they do in the case of explanatory variables (page 8, lines 179-180). Moreover, the authors perform a sensitivity analysis with the entire dataset (with and without missing data), which, in my opinion, would make more sense if the main dataset consisted of individuals with complete data, and the sensitivity analysis with all individuals with imputation of missing data. | We appreciate this suggestion and have revised our approach. “Don’t know” responses are now treated as missing data rather than being interpreted as the absence of disease. Additionally, we have simplified our analysis to focus on complete case analysis only, as the proportion of missing data was minimal. |
| 13 | Statistical methods  In the first paragraph, the authors state that they use STATA software to conduct the statistical analysis. I recommend making this comment at the end of the statistical methodology section, not at the beginning. | We have moved the mention of STATA software to the end of the Statistical Methods section, as suggested. |
| 14 | At the end of page 9 and the beginning of page 10, the authors say they adjust 5 multilevel models. I recommend replacing the term "produced" with "fitted". | We have replaced the term “produced” with “fitted” to align with standard statistical terminology. |
| 15 | As the authors correctly mention, the adjustment of these 5 models, as generated, allows for explaining the effect of the three levels analyzed: individuals, neighborhoods, and provinces. However, it does not allow for an accurate analysis of which risk factors are most relevant, which is one of the study's objectives. For example, different models could be fitted, starting from a baseline model with age and sex, and then adding different groups of explanatory variables to contrast which are significant and which are not. | We agree with the reviewer’s suggestion and have simplified our modeling approach. We now present only two models:  1. An unconditional model (empty model) to assess variance partitioning.  2. A fully adjusted model with a minimal set of relevant variables selected using LASSO regression. |
| 16 | The final model contains 23 covariates with 50 associated coefficients. I consider that, without previously evaluating whether each and every variable is significant, there is a high risk of overfitting and multicollinearity. There are different techniques to reduce dimensionality, such as LASSO techniques, stepwise adjustment, or comparing Bayesian (BIC) or Akaike (AIC) criteria between nested models. I suggest applying some of these techniques to find which variables are most relevant and what their final effect is. | We have addressed this concern by applying LASSO regression for variable selection.  “Model Development and Variable Selection  To reduce the dimensionality of the model and avoid overfitting, we employed LASSO (Least Absolute Shrinkage and Selection Operator) regression for variable selection[20]. LASSO is a penalized regression technique that shrinks the coefficients of less important variables to zero, thereby selecting only the most relevant predictors. This approach is particularly useful when dealing with a large number of covariates, as it minimizes multicollinearity and improves model interpretability. The LASSO model was fitted using the EBIC (Extended Bayesian Information Criterion) to select the optimal lambda value, which balances model fit and complexity. The final model included the following variables, selected based on their contribution to explaining the variance in multimorbidity: Age category, sex, wealth index, education attainment, marital status, ethnicity, body mass index, workplace smoke exposure, new media access and region. The LASSO regression results indicated that these variables were the most significant predictors of multimorbidity, with minimal loss of predictive accuracy” |
| 17 | I have some doubts regarding the use of the multilevel model, particularly concerning the inclusion of the third level: province. Although I am not an expert in the matter, the multilevel methodology does not seem to apply at the provincial level. A typical example of applying multilevel techniques occurs when we have a sample of units at the level in question (in this case, provinces) and we are not interested in analyzing the differences between these units, but we understand that they may explain part of the total variability of the analysis (Harvey Goldstein. Multilevel Statistical Models. Fourth Edition. Page 17). However, in this work, the opposite situation occurs: information is available for all provinces of South Africa (9), and one of the objectives is to analyze the distribution of multimorbidity. Therefore, in my opinion, it would be more advisable to use the province as a fixed effect rather than a random effect. Moreover, the results, as the authors themselves comment, do not seem to support much the contribution of the provincial level. For all these reasons, I would recommend simplifying the multilevel model to two levels, individuals and neighborhoods, and using the province as a fixed effect. | We agree with the reviewer’s recommendation and have simplified the multilevel model to two levels: individuals and neighborhoods. Province is now included as a fixed effect rather than a random effect. This change aligns with the study’s objectives and ensures a more appropriate use of multilevel modeling. |
| 18 | RESULTS  Fixed effects (measures of association)  I suggest modifying the title of this section to "Measures of association: fixed effects". One of the objectives is to determine the risk factors for presenting multimorbidity. Therefore, I would give more relevance to this fact, rather than to the type of adjusted model: with fixed and random effects. Although I recognize this is a very personal opinion. | We have revised the section title to “Measures of Association: Fixed Effects” to better reflect the focus on risk factors for multimorbidity. |
| 19 | I find the writing of this section too dense, and in some cases, in unusual language. For example, on page 13 line 287, the authors say "Men had 0.54 times the odds of being multimorbid..." when it would be more comprehensible to say that the OR in men is 0.54, or that the probability of presenting multimorbidity is lower in men than in women (adding the values and/or confidence intervals in parentheses). Moreover, some of the results show very low significance, including unexpected or anomalous results, such as having a certain level of education being a risk factor compared to having no education. It is for this reason that I recommend adjusting different models, using dimension reduction techniques, to see which is the best final model, which variables have the most impact, and in what direction. My opinion is that with 23 covariates there may be high multicollinearity. | We have completely rewritten the Results section to improve clarity and readability. Key changes include:  - Using simpler language to describe odds ratios (e.g., “Men had lower odds of multimorbidity compared to women [OR = 0.54, 95% CI: 0.44–0.60]”).  - Removing redundant or overly technical phrasing.  - Highlighting only the most significant and interpretable results, as identified through LASSO regression.  These revisions ensure the results are presented in a clear and accessible manner. |
| 20 | On the other hand, on page 13, line 290, they refer to the interpretation of age in terms of a 10-year increment, while Table 3 says that 5-year groups are used (not 10). | We have corrected this inconsistency. |
| 21 | Table 1: characteristics of study population  I recommend adding a column with the p-values of the effect for each analyzed covariate. | We have added a p-value column to Table 1 to show the statistical significance of each covariate’s association with multimorbidity. |
| 22 | I also recommend putting the percentages by rows and not by columns, this would allow seeing for each category the percentage of cases with multimorbidity. For example, in the case of education, it would be seen that the percentage of cases with multimorbidity in the group without studies is 65%, in the case of having primary studies the percentage with multimorbidity is 57%, and in the case of having secondary and higher studies the percentage is 39% and 34% respectively. This bivariate descriptive result would indeed show an expected result: the higher the level of education, the lower the presence of multimorbidity. | We have revised Table 1 to present row percentages instead of column percentages. This change allows readers to see the proportion of individuals with multimorbidity within each category of the covariates. |
| 23 | I also recommend putting all categories of each variable. For example, in the case of tobacco exposure at work, only the values of those who are exposed appear. | We have ensured that all categories of each variable are included in Table 1. For example, both “exposed” and “not exposed” categories are now shown for exposure to workplace smoke |
| 24 | From the results, we can see that in some variables there is a high number of groups, some of which with few cases. It might be advisable to group categories, which would reduce the number of coefficients when adjusting the models. | We have grouped categories for variables with a high number of groups or small sample sizes. |
| 25 | Table 3: Multilevel logistic regression models  The results of Table 3 show that the effect of many of the variables introduced in the model is not significant, and in some cases the direction of the effect is surprising. Introducing variables that are not significant may be affecting the estimation of the rest. It is for this reason that I recommend reducing the dimension of the model. | We have addressed this concern by applying LASSO regression for variable selection. The final model now includes only the most significant variables, reducing the risk of overfitting and multicollinearity. The revised Table 3 reflects this streamlined approach. |
| 26 | A result that I find very interesting is that of tobacco exposure at work. If this effect is maintained by reducing the dimension of the model, it could be an interesting topic to address in the discussion, for example, to propose preventive measures. | We agree that the association between exposure to workplace smoke and multimorbidity is an important finding. This result has been retained in the reduced model and is now discussed in the Discussion section, with a focus on potential preventive measures. |
| 27 | I don't see in Table 3 the results of the variance at the individual level. Typically, if there are three levels, the results of the variance for each of them are shown. But Table 3 only shows the estimates of variances in two of the three levels (for neighborhood and province). | We appreciate the reviewer’s insightful comment regarding the absence of individual-level variance in Table 3. This is an important point, and we would like to clarify why individual-level variance is not typically reported in multilevel logistic regression models with binary outcomes.  In multilevel models with binary outcomes, such as logistic regression, the individual-level variance is not explicitly estimated or reported because it is inherently determined by the nature of the outcome variable. Binary outcomes follow a Bernoulli distribution, where the variance is directly tied to the probability of the outcome. Specifically, for a binary outcome with probability p, the variance is calculated as p(1-p). This means that the individual-level variance is not a separate parameter to be estimated but is instead a function of the predicted probabilities generated by the model.  Furthermore, in logistic multilevel models, the residual variance at the individual level is fixed to approximately 3.29 to account for the underlying assumptions of the logistic model. This fixed value arises because logistic regression assumes that the error terms follow a logistic distribution, and the variance of this distribution is mathematically defined as pi squared divide by 3 . This constraint ensures that the model remains identifiable and allows for the separation of individual-level variability from the variances at higher levels, such as neighborhoods or provinces.  In summary, the individual-level variance is not reported in multilevel logistic regression models because it is inherently determined by the Bernoulli distribution of the binary outcome and is fixed to pi squared divide by 3 to maintain model identifiability. This approach allows us to focus on estimating and interpreting the variances at higher levels, which are of primary interest in multilevel analyses.  References:  - Goldstein, H. (2011). Multilevel Statistical Models (4th ed.). Wiley.  - Snijders, T. A. B., & Bosker, R. J. (2012). Multilevel Analysis: An Introduction to Basic and Advanced Multilevel Modeling (2nd ed.). Sage. |
| 28 | DISCUSSION  On page 18 lines 403-404 the authors say "The relationship between increasing education up to secondary level and reduced odds of multimorbidity..." when in my opinion it is the opposite (according to the multivariate model). In Table 3 the OR for secondary education is 1.31, and the reference category is cited as equivalent to having no studies. This is a surprising result, which is probably affected by multicollinearity and/or possible overfitting. | We have removed this statement from the Discussion section. In the revised model, the association between education level and multimorbidity is no longer significant, likely due to the reduction in multicollinearity and overfitting achieved through LASSO regression. |
| 29 | RECOMMENDATIONS  My main recommendation, oriented towards the objective of determining risk factors for presenting multimorbidity, would be to reduce the dimension of the final model. From the results of Table 3, it seems that some of the introduced variables are not significant. And given the high number of coefficients in the final model, the estimation of effects may be inefficient.  For this purpose, different techniques can be used, such as stepwise techniques, or the comparison of model performance based on measures that penalize the introduction of additional coefficients: such as the BIC.  I also recommend introducing the effect of provinces as a fixed effect, and reducing the random effects to individuals and neighborhoods. | We have addressed these recommendations as follows:  1. Reduced the dimension of the final model using LASSO regression, retaining only significant variables.  2. Introduced provinces as a fixed effect and simplified the multilevel model to two levels: individuals and neighborhoods.  3. Removed non-significant variables to improve the efficiency and interpretability of the model.  Summary of Changes  - Added p-values and row percentages to Table 1.  - Included all categories for each variable in Table 1.  - Grouped categories for variables with small sample sizes.  - Reduced the number of variables in the final model using LASSO regression.  - Retained and discussed the association between tobacco exposure at work and multimorbidity.  - Clarified the handling of individual-level variance in multilevel logistic regression.  - Removed inconsistent statements from the Discussion section.  - Simplified the multilevel model to two levels and included provinces as a fixed effect.  We believe these revisions have significantly improved the manuscript and addressed all reviewer comments. Thank you for your valuable feedback. |
