# Supplementary material for: Prevalence, patterns, and determinants of multimorbidity in South Africa: Insights from a nationally representative survey
Source: PLOS Glob Public Health. 2025 May 9;5(5):e0004603. doi: 10.1371/journal.pgph.0004603 (PMC12063843; doi:10.1371/journal.pgph.0004603)
Supplement: Attachment — Submitted filename: 06_ResponsesToComments_auresp_2.docx [file pgph.0004603.s005.docx]

|  | Comment | Suggest response |
| --- | --- | --- |
| 1 | 1. Reading the updated version, I only detected one minor error: on page 11, line 251, the authors say "Women had significantly higher odds of multimorbidity compared to women", when I understand it should say "Women had significantly higher odds of multimorbidity compared to men". | Thank you for pointing this mistake out. This has been corrected to "Women had significantly higher odds of multimorbidity compared to men". |
