# Supplementary material for: Prevalence, patterns, and determinants of multimorbidity in South Africa: Insights from a nationally representative survey
Source: PLOS Glob Public Health. 2025 May 9;5(5):e0004603. doi: 10.1371/journal.pgph.0004603 (PMC12063843; doi:10.1371/journal.pgph.0004603)
Supplement: Attachment — Submitted filename: 06_ResponsesToComments_auresp_3.docx [file pgph.0004603.s006.docx]

|  | Comment | Suggest response |
| --- | --- | --- |
| 1 | The Abstract must be structured and conceptually divided into sections, including Background, Methodology, Principal Findings, and Conclusions/Significance. Please consider this in the manuscript. | Thank you for pointing this out, we have formatted the abstract into sections and incorporated minor edits to increase the conceptual division into sections. |
| 2 | The title of the paper should contain a specific context where the study was conduted. A resource-limited setting appears too broad and should replaced by a specific context. | We appreciate this comment and have changed the title to be more context specific:  Prevalence, Patterns, and Determinants of Multimorbidity in South Africa: Insights from a Nationally Representative Survey |
